# Sub-chronic stress exerts partially distinct behavioral and epigenetic effects in male and female mice

**DOI:** 10.3389/fnbeh.2025.1649660

**Published:** 2025-09-17

**Authors:** Matthew J. Domanico, Sophie Stevens, Iris Wainston, Emily Khoo, Corey McCall, Benjamin D. Swack, Benjamin D. Sachs

**Affiliations:** Department of Psychological and Brain Sciences, Villanova University, Villanova, PA, United States

**Keywords:** light–dark emergence, open field, effort-related reward choice tests, prodynorphin, inhibitory kappa B kinase beta genes, NEMO, D2 dopamine receptor genes

## Abstract

**Introduction:**

Stress-related disorders, such as major depression, anxiety disorders, and post-traumatic stress disorder, lead to considerable disease burden and are notoriously difficult to treat. These disorders are characterized by striking sex differences, but the neurobiological underpinnings of the disparities in mental health between men and women remain largely undefined. With an improved understanding of the biological factors that promote or protect against psychopathology, it may become possible to design interventions that enhance resilience. Preclinical research using rodent models can provide fundamental insight into potential sex differences in the neurobiological consequences of stress, which could have important implications for our understanding of stress-related disorders.

**Methods:**

Towards this end, the current work compared stress-induced alterations in DNA methylation and behavior in male and female c57BL/6 mice. A subchronic stress paradigm consisting of five days of mild stressors was used, and behavioral outcomes were assessed using the elevated plus maze and the light-dark emergence, open field, forced swim and effort-related reward choice tests.

**Results:**

Statistical analyses using two-way ANOVAs revealed that although some of the effects of stress in the light-dark emergence test were specific to females, both sexes were susceptible to several behavioral consequences of this stress paradigm. Stress was also shown to decrease global DNA methylation in the nucleus accumbens one week following the end of stress exposure in both sexes, but no significant effects were observed two hours following stress. In the hippocampus, no global DNA methylation differences were observed at either time point. Targeted evaluations using methylation-specific PCR revealed sex differences in stress-induced changes in DNA methylation at sites in the prodynorphin and inhibitory kappa B kinase beta genes in the nucleus accumbens. In contrast, no significant sex-by-stress interactions were observed for methylation changes in the hippocampus, although stress significantly increased DNA methylation of prodynorphin and inhibitory kappa B kinase beta two hours after the final stress exposure and reduced methylation of the NEMO and D2 dopamine receptor genes one week following stress.

**Discussion:**

Overall, these findings provide further evidence of sex differences in stress susceptibility and suggest that sex differences in epigenetic adaptations to stress could contribute to the partially distinct behavioral outcomes of stress in males and females.

## 1 Introduction

Major depression and anxiety disorders are highly prevalent, debilitating conditions that exert a disproportionate toll on women (Seedat et al., 2009). The basis of this mental health disparity is likely multifactorial, but sex differences in stress susceptibility and stress exposure are considered major contributing factors (Bangasser and Cuarenta, 2021; Farhane-Medina et al., 2022). Using animal models to compare the behavioral and neurobiological responses to stress in males and females could provide new insight into the biological mechanisms underlying this heightened sensitivity of females to stress-related disorders and could help uncover sex-specific molecular adaptations to stress (Bangasser and Wicks, 2017). Ultimately, this type of knowledge could pave the way for more personalized treatments that target the unique stress-induced molecular pathology in males and females. Indeed, significant sex differences have been reported in the efficacy of treatments for both stroke (Sohrabji et al., 2017) and glioblastoma (Yang et al., 2019) in males compared to females, and it has been argued that the failure to include sex as a biological variable in both clinical and preclinical studies of stress-related disorders may contribute to the relative lack of progress in developing novel therapeutics for these conditions (Galea et al., 2020). Importantly, the current study not only includes both males and females, but it also specifically examines sex as a biological variable, a still-too-uncommon practice that is required to determine whether stress differentially impacts the behavior of males and females (Rechlin et al., 2022; Shansky and Woolley, 2016; Dalla et al., 2024).

Although many preclinical studies involving stress responses have focused exclusively on males (Mancini et al., 2025), prior reports suggest that a six-day sub-chronic variable stress (SCVS) paradigm is sufficient to induce behavioral dysfunction in female mice, but not males (Hodes et al., 2015; LaPlant et al., 2009; Williams et al., 2020; Zhang et al., 2018), suggesting that sub-chronic stress paradigms could be useful in identifying mechanisms underlying sex differences in stress responses. Several cellular and molecular mechanisms have been suggested to contribute to the heightened sensitivity of females to SCVS. For example, both sex differences in neurophysiological adaptations (Zhang et al., 2018; Brancato et al., 2017) and epigenetic responses (Hodes et al., 2015) to SCVS have been implicated in the increased vulnerability of females. Prior work using a five-day stress (5DS) model consisting of forced swimming, restraint, and tail suspension reported that stress increases the expression of DNA methylase 3a (Dnmt3a) in the nucleus accumbens (NAc) of females but not males (Baugher et al., 2022). However, several other studies have shown that stress can also increase Dnmt3a expression in males. For example, exposure to foot shock stress increases Dnmt3a expression in the hippocampus (Hip) of male rats (Sales and Joca, 2018), while social defeat stress (SDS) has been reported to increase the expression of Dnmt3a in the NAc (LaPlant et al., 2010). Even SCVS, which generally induces behavioral effects in females but not males, has been reported to increase Dnmt3a expression in the NAc in both sexes (Hodes et al., 2015). Given prior reports of stress impacting Dnmt3a in both the NAc and the Hip, the current work measured DNA methylation in both of these areas.

The potential importance of excessive Dnmt3a expression in regulating stress responses has been supported by studies showing that overexpression of Dnmt3a in the NAc increases depression- and anxiety-like behaviors, and knocking out Dnmt3a induces antidepressant and anxiolytic-like effects in mice exposed to 3 days of stress (Hodes et al., 2015). Similarly, both systemic and intra-hippocampal administration of DNA methylation inhibitors have been shown to induce antidepressant-like effects in rodents (Sales and Joca, 2018; Sales et al., 2011; Sales and Joca, 2016), and the efficacy of Hip-specific treatments provided further rationale for analyzing this structure in addition to the NAc. Together, these findings suggest that sex differences in DNA methylation in either the NAc or the Hip could both have important implications for behavioral outcomes following stress. Prior research suggests that DNA methylation within promoter regions is associated with transcriptional repression whereas methylation of coding regions is associated with transcriptional activation (Wu et al., 2011), and we therefore assessed methylation in both coding and promoter regions where possible.

Given that SCVS has been reported to induce behavioral changes that are highly sex-dependent (Hodes et al., 2015), whereas the closely related 5DS paradigm (which replaces foot shocks from SCVS with forced swimming) has been reported to induce behavioral changes that are largely independent of sex (Baugher et al., 2022), the current study aimed to test whether a third sub-chronic stress paradigm would produce distinct behavioral outcomes in males and females. This third paradigm, called five-day variable stress (5DVS), replaces the forced swimming from 5DS with exposure to fox urine. In addition, in light of the prior work revealing sex differences in Dnmt3a expression following sub-chronic stress (Hodes et al., 2015; Baugher et al., 2022), the present study aimed to compare the epigenetic consequences of the 5DVS model in male and female c57BL/6 mice.

In keeping with previous work in this area, the behavioral consequences of stress were first assessed using a panel of commonly used tests, including the light–dark emergence (LDE), forced swim (FST), novel open field (NOF), and elevated plus maze (EPM) (Hodes et al., 2015; Baugher et al., 2022; Baugher and Sachs, 2022). Although these short-term tests have been reported to be sensitive to sub-chronic stressors and can provide insight into individual differences in stress susceptibility, their interpretation is increasingly controversial (Anyan and Amir, 2018; Commons et al., 2017; Unal and Canbeyli, 2019; Stupart et al., 2023; Gyles et al., 2023) and can be difficult to align with Research Domain Criteria (RDoC)-defined behavioral domains. Consequently, the current study also used a home cage version of an effort-related decision-making task (Matas-Navarro et al., 2023) in which animals had simultaneous free access to a high-effort reward (i.e., a running wheel) and a low-effort reward (a saccharin solution). Effort-based decision-making tasks have been shown to be significantly impacted by blocking dopaminergic neurotransmission (Salamone et al., 1994; Correa et al., 2016), treatment with stimulants (Lopez-Cruz et al., 2024), and administration of corticosterone (Dieterich et al., 2020), but the effects of stress on these tasks have been relatively understudied. In addition to examining behavior, DNA methylation was examined globally and at several candidate genes that had previously been identified as being differentially impacted by stress in males and females (Baugher et al., 2022).

## 2 Materials and methods

### 2.1 Animals

This study used 72 male and 67 female C57BL/6 mice that were bred in-house at Villanova University derived from C57BL/6 J breeding pairs originally obtained from Jackson Laboratories. Three separate cohorts of mice were run for the rapid behavioral tests (~20 mice per cohort), and four cohorts were run in the home cage effort-related choice test (~10 mice per cohort). Two additional cohorts were run for the epigenetics analyses (one at each time point). These ‘epigenetic’ cohorts were subjected to stress, as described below, but were not examined in any behavioral tests. The mice were housed in a temperature- and humidity-controlled room on a 12-h light–dark cycle. For most experiments, mice were group housed with one to four same-sex littermates. However, the home cage effort-related choice (ERC) test requires single housing of all animals. Animals had ad libitum access to food and water except during behavioral testing and exposure to the daily stressors. All mice were between eight and 12 weeks old at the start of experiments. All studies were performed in accordance with protocols approved by the Institutional Animal Care Use Committee (IACUC) at Villanova University and in keeping with the Guide.

### 2.2 Five-day variable stress protocol

The 5DVS protocol was adapted from prior work and consists of exposure to 1 h of stress each day for 5 days. On days one and four, the 5DVS mice were subjected to 1 h of restraint in ventilated 50 mL conical tubes. On days two and five, the 5DVS mice were exposed to the scent of fox urine for 1 h. On day three, the mice in the 5DVS condition were suspended by their tails for 1 h. The restraint and tail suspension stressors are also used in the previously published SCVS and 5DS paradigms, but fox urine exposure is unique to 5DVS, as 5DS uses forced swimming as the third stressor (Baugher et al., 2022) and SCVS utilizes foot shocks as its third stressor (Hodes et al., 2015). Predator odors have been previously used in six-day variable stress studies in rats (Eck et al., 2020). Control mice were briefly handled on each of the 5 days that the 5DVS mice were stressed to control for potential behavioral differences induced by human handling.

### 2.3 Behavioral testing

Behavioral testing using rapid behavioral tests began on day 6 (24 h after the final stress exposure) and continued with one test daily over 4 days. Mice were assessed in the light–dark emergence (LDE) test on day 6, the elevated plus-maze (EPM) on day 7, the open-field test (OFT) on day 8, and the forced swim test (FST) on day 9. The home cage ERC was conducted in separate cohorts of animals starting 3 days before the beginning of stress exposure and continuing for 2 days following the end of stress exposure for a total of 10 days of testing.

#### 2.3.1 Light–dark emergence

The LDE was performed approximately 24 h after the final 5DVS stressor as we have described previously (Baugher et al., 2022). Briefly, the mice were placed into the dark chamber, and their behavior was monitored for 5 min by ANY-maze tracking software, which calculated the distance traveled, time spent, and number of entries in the light chamber, as well as the latency to enter the light compartment.

#### 2.3.2 Elevated plus Maze

The EPM was performed as described previously (Baugher et al., 2022). The distance traveled and time spent in each arm, as well as the latency to enter the open arm, were recorded using ANY-maze animal tracking software for the test duration of 5 min.

#### 2.3.3 Novel open-field test

The novel open-field test was performed on day eight of the paradigm as we have described previously (Baugher and Sachs, 2022). In this test, mice are placed in the corner of a plexiglass container, and their behavior was analyzed for 20 min by ANY-maze tracking software. The overall distance traveled, the distance traveled in the center of the box, and the time spent in the center of the box were recorded.

#### 2.3.4 Forced swim test

The FST was performed as we have described previously (Baugher and Sachs, 2022). The mice were placed in 4-L beakers filled with 2,500 mL of water at 25 °C for 6 min. The distance traveled, time each mouse spent immobile, number of immobile episodes, and the latency to the first immobile episode were recorded using ANY-maze software.

#### 2.3.5 Home cage effort-related choice test

The ERC was conducted by housing a mouse in an oversized cage containing an upright running wheel (8.2 inch diameter, NiteAngel) and two water bottles, one of which contained standard drinking water and the other contained a 0.03% saccharin solution. The number of rotations and the amount of liquid consumed from each water bottle were recorded once daily. Data were collected for 3 days prior to stress exposure, for all 5 days of stress exposure, and for 2 days following the end of stress. During the hour of stress exposure in the 5DVS group, the control mice remained in their home cages with the running wheel and solution bottles removed to ensure they did not have access to either reward while the experimental mice were exposed to the stressors.

### 2.4 Epigenetic analyses

#### 2.4.1 DNA isolation

Mice were euthanized at two time points (2 h or 1 week) after the final stress exposure in 5DVS. The control mice were sacrificed at the same time points, and the mice were age-matched and sex-matched. Extracta Plus DNA (Quantabio, Beverly, MA) kits were used to purify the genomic samples according to the manufacturer’s instructions.

#### 2.4.2 Methylation-specific PCR

Purified genomic DNA was divided into three aliquots: one of which remained undigested, the second was digested using HpaII, and the third was digested with MspI. HpaII cuts unmethylated, but not methylated CpG sites, whereas Msp1 cuts CpG sites regardless of their methylation status. To quantify methylation, real-time quantitative polymerase chain reaction (rt-qPCR) was performed in duplicate. The master mix for each brain region consisted of PerfeCTa SYBR® Green FastMix (Quantabio), forward and reverse primers, and water. Real-time PCR was performed on a StepOne plus instrument using StepOne software. Melting curves of all PCR runs were analyzed to ensure the proper number of products (dependent on the number of HpaII/MspI binding sites). A modified version of the ΔΔCT method was used to analyze the methylation levels (Ni et al., 2019) using the following formula to determine percent methylation:


$$\left[2^{\wedge }(-\Delta \mathrm{CT}(\text{HpaII}))-2^{\wedge }(-\Delta \mathrm{CT}(\text{MspI}))*100\%\right]$$

Any negative values were considered to be 0%. A list of primers used can be found in Table 1.

**Table 1:** Primer sequences used for the epigenetic analysis.

Gene	Forward primer	Reverse primer
NEMO	ATAGGAGTGCCTGGCTGTTAG	AGCTTCTCCAAGCTCAGTCTC
NEMO Promoter	AGAGTATGGCACTTTGGGGTTT	AGTCCTAGTCAGGCGGTCA
IKKβ	GCATCGATATGAGCTGGTCAC	AGAAAGCTCACCCACCTTCCT
PDYN	AGCTGCCTAGGCTCTGTAAGT	TGGTTGTCCCACTTCAGCTT
PDYN Promoter	AAGTGGCCGCATTGAAAGTG	GGCCCGAGTGAGACACAATA
DRD2	TGGAGCCAAAAGCAGTCTGT	GCCATCCTTCAGGTTTCCGA
Global Methylation	N/A	N/A

#### 2.4.3 Global methylation analysis

Global methylation analysis was performed using Epigentek’s MethylFlash Global DNA Methylation (5-mC) ELISA Easy Kits (Epigentek, East Farmingdale, NY) according to the manufacturer’s instructions.

### 2.5 Statistical analyses

Statistical analyses were performed using SPSS software (IBM, version 29). Most data were analyzed using two-way ANOVAs with two between-subjects fixed factors of sex (male or female) and stress (5DVS or control). The home cage ERC included the same two between-subjects factors but also included a within-subjects, repeated measures analysis. The sphericity of repeated measures data was analyzed using Mauchly’s Test of Sphericity. When the assumption of sphericity was violated (*p* < 0.05), the degrees of freedom were adjusted using the Greenhouse–Geisser correction.

## 3 Results

### 3.1 Behavioral results

#### 3.1.1 Light–dark emergence

**Figure 1:**
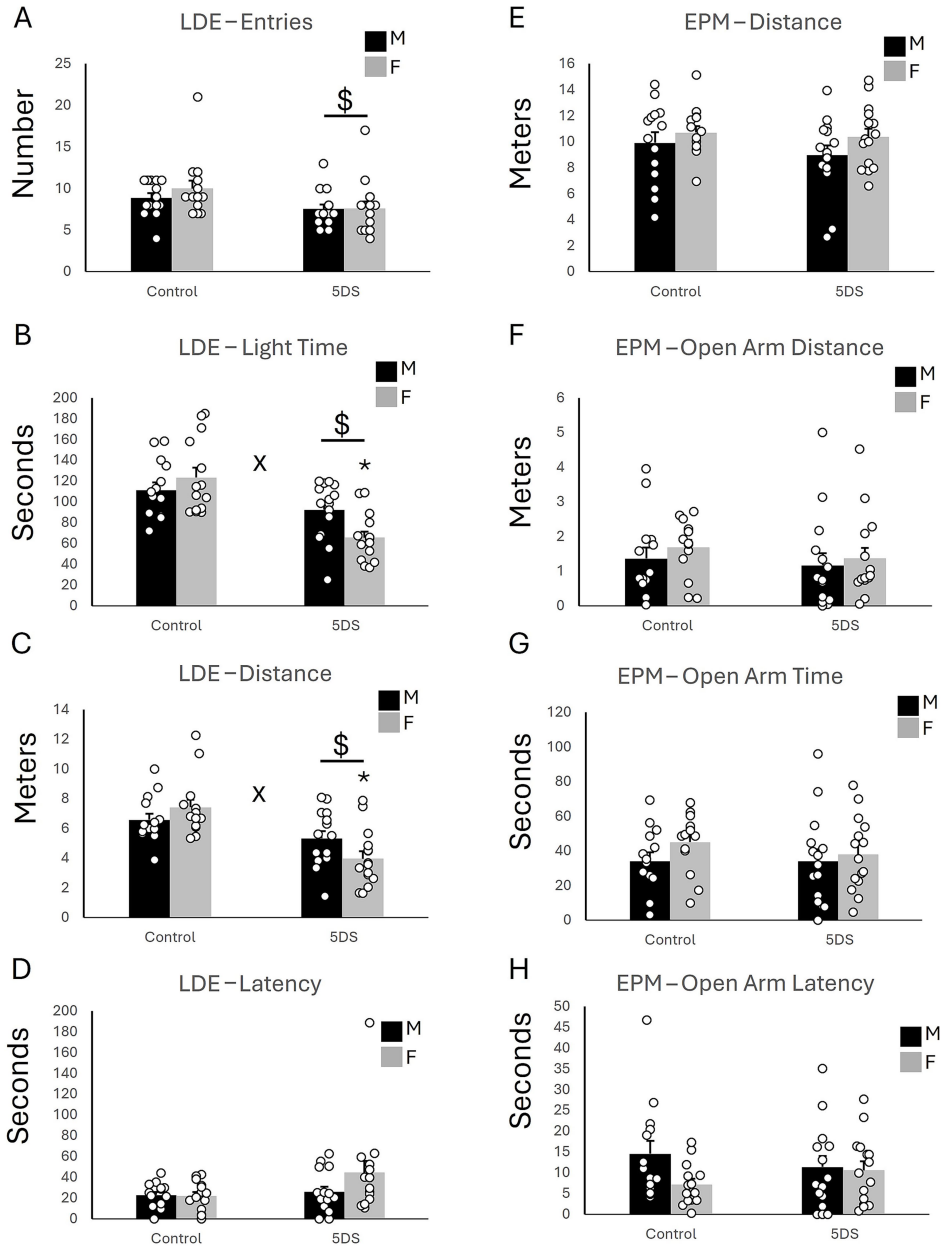
The effects of stress in the light–dark emergence and elevated plus maze tests. **(A)** The number of entries into the light chamber in the LDE. **(B)** The time spent in the light chamber of the LDE. **(C)** The distance traveled in the light chamber of the LDE. **(D)** The latency to first enter the light chamber of the LDE. **(E)** The total distance traveled in the EPM. **(F)** The distance traveled in the open arms of the EPM. **(G)** The amount of time spent in the open arms of the EPM. **(H)** The latency to first enter an open arm of the EPM. ‘$’ indicates a significant main effect of stress, and ‘x’ indicates a significant stress by sex interaction by two-way ANOVA (*p* < 0.05). *n* = 14 per sex in the control groups and 15 per sex in the 5DVS groups.

The LDE revealed a significant main effect of stress on light entries [*F*_(1,54)_ = 6.232, *p* = 0.016, Figure 1A], time spent in the light compartment [*F*_(1,54)_ = 25.801, *p* < 0.001; Figure 1B], and distance traveled in the light compartment [*F*_(1,54)_ = 23.654, *p* < 0.001; Figure 1C]. However, the effects of stress on light latency were not statistically significant [*F*_(1,54)_ = 3.547, *p* = 0.065; Figure 1D]. Mice in the stress-exposed group entered the light compartment less, spent less time in the light compartment, and traveled less than their unstressed counterparts. In addition, significant stress-by-sex interactions were observed for time spent in the light [*F*_(1,54)_ = 6.481, *p* = 0.014; Figure 1B] and distance traveled in the light compartment [*F*_(1,54)_ = 4.129, *p* < 0.047; Figure 1C], but not for light entries [*F*_(1,54)_ = 0.521, *p* = 0.474; Figure 1A] or light latency [F_(1,54)_ = 2.002, *p* = 0.163; Figure 1D]. *Post hoc* analyses revealed that the effects of 5DVS on light time [*F*_(1,54)_ = 29.234, *p* < 0.001; Figure 1B] and light distance [*F*_(1,54)_ = 25.464, *p* < 0.001; Figure 1C] were only significant in females, not males. No significant main effects of sex were observed on light entries [*F*_(1,54)_ = 0.657, *p* = 0.421; Figure 1A], light time [*F*_(1,54)_ = 0.942, *p* = 0.336; Figure 1B], light distance [*F*_(1,54)_ = 0.303, *p* = 0.584; Figure 1C], or light latency [*F*_(1,54)_ = 1.68, *p* = 0.2; Figure 1D].

#### 3.1.2 Elevated plus maze

No significant main effects of stress were observed in the EPM for the total distance traveled [*F*_(1,54)_ = 0.829, *p* = 0.367; Figure 1E], the distance traveled in the open arms [*F*_(1,54)_ = 0.485, *p* = 0.489; Figure 1F], the time spent in the open arms [*F*_(1,54)_ = 0.159, *p* = 0.691; Figure 1G], and the latency to enter the open arms [*F*_(1,54)_ = 0.003, *p* = 0.960; Figure 1H]. Similarly, no significant main effects of sex were observed in the EPM for the total distance traveled [*F*_(1,54)_ = 2.5, *p* = 0.12; Figure 1E], the distance traveled in the open arms [*F*_(1,54)_ = 0.507, *p* = 0.48; Figure 1F], the time spent in the open arms [*F*_(1,54)_ = 1.275, *p* = 0.264; Figure 1G], and the latency to enter the open arms [*F*_(1,54)_ = 2.807, *p* = 0.10; Figure 1H]. No significant stress-by-sex interactions were observed in the EPM for the total distance traveled [*F*_(1,54)_ = 0.199, *p* = 0.657; Figure 1E], the distance traveled in the open arms [*F*_(1,54)_ = 0.112, *p* = 0.74; Figure 1F], the time spent in the open arms [*F*_(1,54)_ = 0.739, *p* = 0.394; Figure 1G], and the latency to enter the open arms [*F*_(1,54)_ = 1.849, *p* = 0.18; Figure 1H].

#### 3.1.3 Open field test

**Figure 2:**
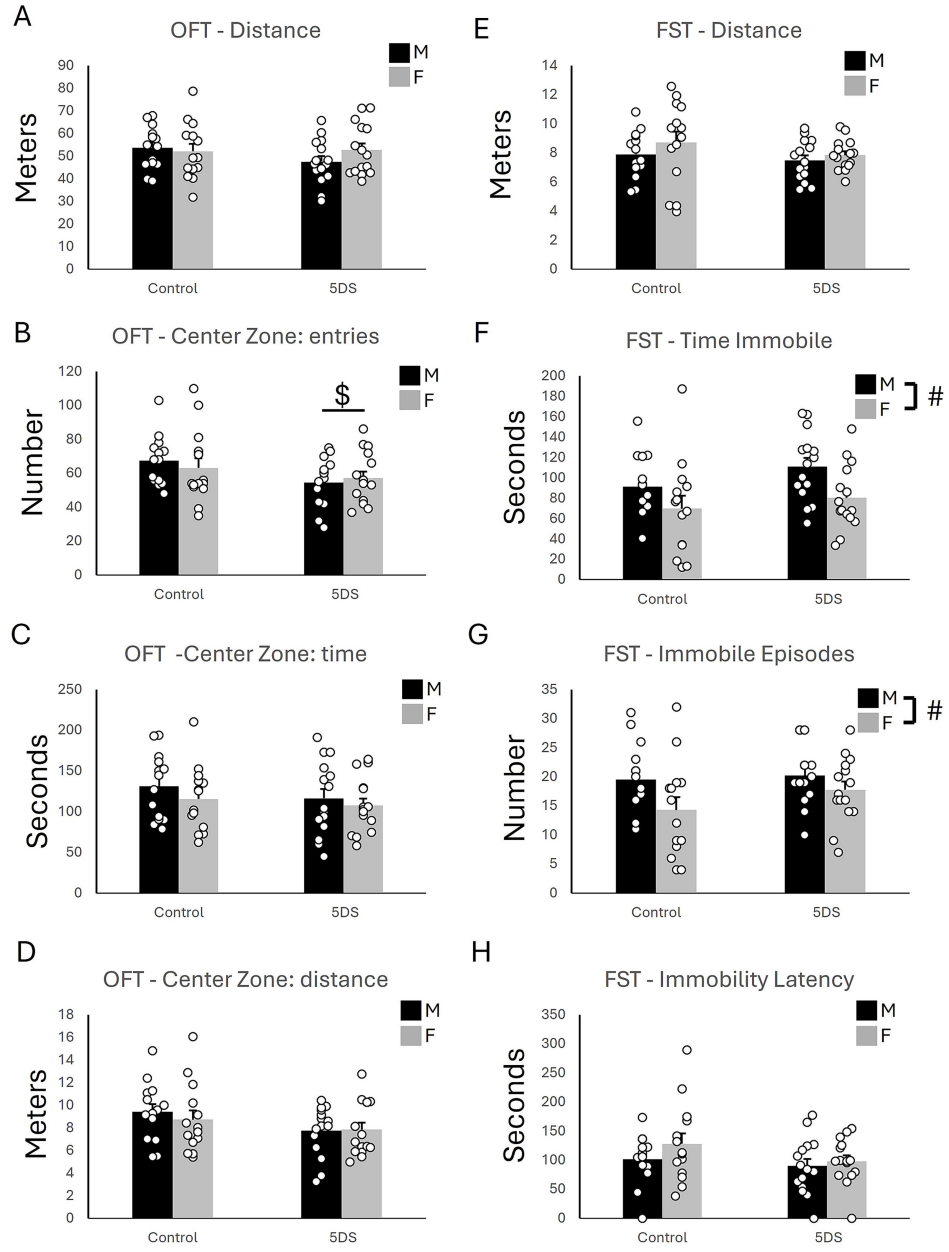
The effects of stress in the open field and forced swim tests. **(A)** The total distance traveled in the open field. **(B)** The number of entries into the center of the open field. **(C)** The time spent in the center of the open field. **(D)** The distance traveled in the center of the open field. **(E)** The distance swam in the forced swim test. **(F)** The time spent immobile in the forced swim test. **(G)** The number of immobile episodes in the forced swim test. **(H)** The latency until the first immobile episode in the forced swim test. ‘$’ indicates a significant main effect of stress, and ‘#’ indicates a significant main effect of sex by two-way ANOVA (*p* < 0.05). *n* = 14 per sex in the control groups and 15 per sex in the 5DVS groups.

In the OFT, no significant effects of 5DVS were observed on total distance traveled [*F*_(1,54)_ = 0.943, *p* = 0.336; Figure 2A] or the distance traveled in the center [*F*_(1,54)_ = 3.525, *p* = 0.066; Figure 2D]. However, a main effect of 5DVS was observed on the number of center zone entries [*F*_(1,54)_ = 4.680, *p* = 0.035; Figure 2B], in which stress reduced the number of center zone entries in both male and female mice. However, no significant main effect of 5DVS was found in the time spent in the center zone [F_(1,54)_ = 1.160, *p* = 0.286; Figure 2C]. No significant effects of sex were observed on total distance traveled [*F*_(1,54)_ = 0.414, *p* = 0.523; Figure 2A], the number of center zone entries [*F*_(1,54)_ = 0.034, *p* = 0.854; Figure 2B], the time spent in the center zone [*F*_(1,54)_ = 1.359, *p* = 0.249, Figure 2C], or the distance traveled in the center [*F*_(1,54)_ = 0.192, *p* = 0.663, Figure 2D]. Finally, no significant stress-by-sex interactions were observed on the total distance traveled in the OFT [*F*_(1,54)_ = 1.43, *p* = 0.237, Figure 2A], the number of center zone entries [*F*_(1,54)_ = 0.654, *p* = 0.422, Figure 2B], the time spent in the center zone [*F*_(1,54)_ = 0.10, *p* = 0.753, Figure 2C], or the distance traveled in the center [*F*_(1,54)_ = 0.315, *p* = 0.577; Figure 2D].

#### 3.1.4 Forced swim test

In the FST, no significant main effects of stress [*F*_(1,54)_ = 1.779, *p* = 0.188] or sex [*F*_(1,54)_ = 1.437, *p* = 0.236] on total distance were observed (Figure 2E), and the stress-by-sex interaction was also not significant [*F*_(1,54)_ = 0.216, *p* = 0.644]. However, a main effect of sex was observed for the time immobile [*F*_(1,54)_ = 7.410, *p* = 0.009; Figure 2F] in which female mice spent less time immobile than male mice. No significant effect of stress was observed for time immobile [*F*_(1,54)_ = 2.525, *p* = 0.118], and the stress-by-sex interaction was not significant [*F*_(1,54)_ = 0.218, *p* = 0.643]. A main effect of sex was also observed for the number of immobile episodes [*F*_(1,54)_ = 5.215, *p* = 0.026; Figure 2G] in which female mice had fewer immobile episodes than the male mice. As for time immobile, no significant main effect of stress [*F*_(1,54)_ = 1.521, *p* = 0.223] and no stress-by-sex interaction [*F*_(1,54)_ = 0.667, *p* = 0.418] were observed for the number of immobile episodes (Figure 2G). No significant main effects of stress [*F*_(1,54)_ = 2.507, *p* = 0.119] or sex [*F*_(1,54)_ = 1.707, *p* = 0.197] on the latency to the first immobility were observed (Figure 2H), and the stress-by-sex interaction was also not significant [*F*_(1,54)_ = 0.464, *p* = 0.499].

#### 3.1.5 Effort-related choice behavior in the home cage

**Figure 3:**
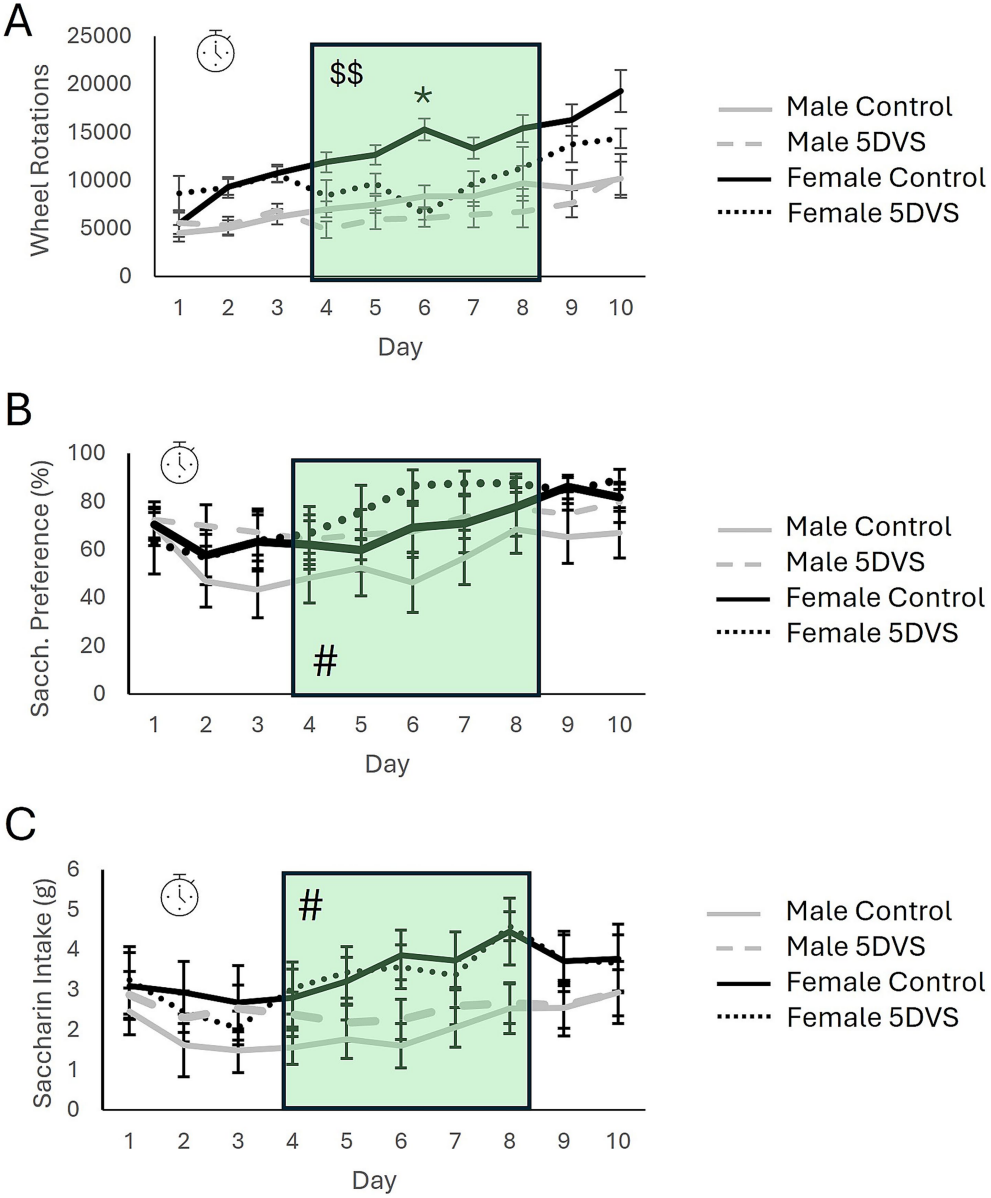
The effects of stress in the effort-related reward choice test. **(A)** Wheel running behavior of mice over the 10-day experiment. **(B)** Saccharin preference over the 10-day experiment. **(C)** Total saccharine intake over the 10-day experiment. The green shading highlights the days on which stress was applied. The clock symbol indicates a significant within-subjects effect of time by repeated measures analysis. The ‘$$’ indicates a significant main effect of stress when only data from stress-exposure days are analyzed. The ‘*’ in A indicates a significant difference between the control and 5DVS-exposed groups on day 6.

In the ERC, when wheel-running behavior was measured, there was a significant overall main effect of time in which animals increased their running progressively across days [*F*_(4.341, 37)_ = 17.756, *p* < 0.001; Figure 3A]. Post-hoc analyses revealed that mice ran significantly more on days 3 & 5–10 compared to their running on day 1 (*p*’s < 0.05). A significant stress-by-time interaction was also observed where control mice increased their running significantly more compared to stress-exposed mice over the course of the experiment [*F*_(4.341, 37)_ = 4.874, *p* < 0.001; Figure 3A]. Further, post-hoc analyses revealed a significant effect of stress on day 6 where control mice ran significantly more than stress-exposed mice (*p* < 0.05; Figure 3A). There was also a significant sex-by-time interaction where females increased their running behavior significantly more than males over time regardless of exposure to stress [*F*_(4.341, 37)_ = 3.1184, *p* = 0.014; Figure 3A]. This effect appeared to be driven by control females, as running was increased in this group on days 3 and 5–10 (*p*’s < 0.05) compared to day 1, but there were no time points at which stressed females ran significantly more than they did on day 1. However, the sex-by-stress-by-time interaction was not statistically significant [*F*_(4.341, 37)_ = 1.428, *p* = 0.226; Figure 3A].

When running behavior was analyzed exclusively on the days in which stress was applied, between-subjects analysis revealed a significant overall main effect of stress in which mice exposed to stress ran less than control mice [*F*_(1, 37)_ = 6.358, *p* = 0.017; Figure 3A]. A significant main effect of sex was also observed for this five-day period [*F*_(1, 37)_ = 24.255, *p* < 0.001; Figure 3A], but the stress-by-sex interaction was not significant [*F*_(1, 37)_ = 0.103, *p* = 0.75; Figure 3A]. Additionally, repeated measures analysis revealed a significant within-subjects effect of time during the 5 days of stress exposure in which mice increased their running across days [*F*_(2.068, 37)_ = 6.300, *p* = 0.003].

Within the ERC, saccharin consumption and preference were recorded in addition to wheel running. For saccharin preference, there was a significant main effect of time [*F*_(3.102, 30)_ = 4.083, *p* = 0.009; Figure 3B]. When pre- and post-stress days were included in the analysis, between-subjects analysis revealed no significant effects of sex [*F*_(1, 30)_ = 3.625, *p* = 0.068; Figure 3B] or stress [*F*_(1, 30)_ = 1.690, *p* = 0.093; Figure 3B], and no significant interaction [*F*_(1, 30)_ = 0.009, *p* = 0.925; Figure 3B]. However, when data were analyzed for between-subjects effects on the 5 days when stress was applied, there was a significant overall main effect of sex [*F*_(1,30)_ = 6.019, *p* = 0.021] where females preferred saccharin more than males during the stress-exposure period (Figure 3B). However, no significant main effect of stress was observed [*F*_(1,30)_ = 1.993, *p* = 0.169], and the stress-by-sex interaction was also not significant [*F*_(1,30)_ = 0.058, *p* = 0.812]. Regarding total saccharin consumption, a significant main effect of time was observed that largely mimicked the pattern observed for saccharin preference [*F*_(3.517, 30)_ = 4.455, *p* = 0.004; Figures 3B,C]. Between-subjects analysis revealed a significant main effect of sex in which female mice drank more saccharin than male mice [*F*_(1,30)_ = 12.047, *p* = 0.002], but the effect of stress was not significant [*F*_(1,30)_ = 0.152, *p* = 0.699], and neither was the stress-by-sex interaction [*F*_(1,30)_ = 1.967, *p* = 0.173; Figure 3C].

### 3.2 Molecular results

#### 3.2.1 Methylation analyses

**Figure 4:**
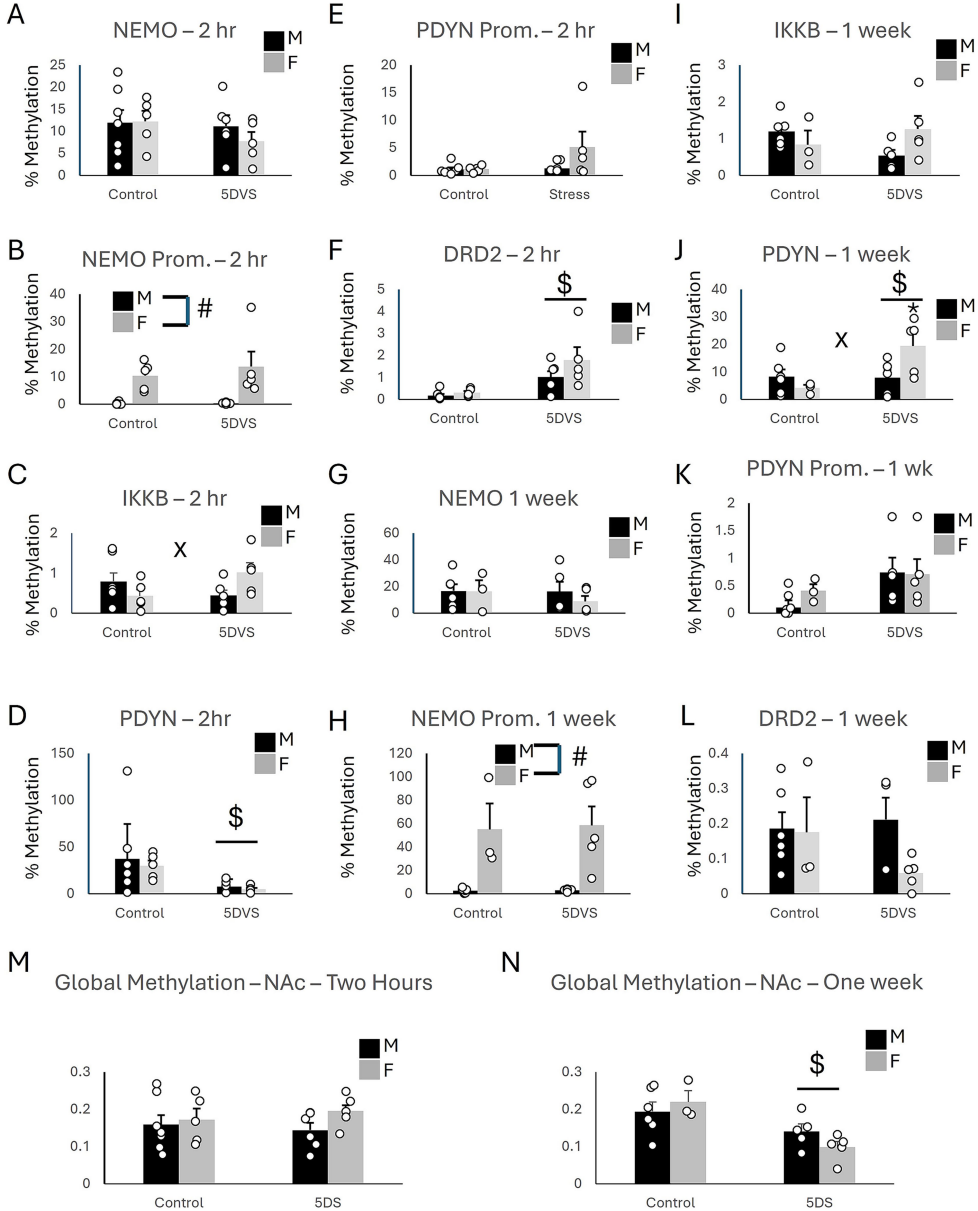
Epigenetic consequences of stress in the nucleus accumbens. **(A)** Methylation of NEMO in the coding region. **(B)** Methylation of NEMO in the promoter region. **(C)** Methylation of IKKβ in the coding region. **(D)** Methylation of PDYN in the coding region. **(E)** Methylation of PDYN in the promoter region. **(F)** Methylation of DRD2 in the promoter region. **(G)** Methylation of NEMO in the coding region. **(H)** Methylation of NEMO in the promoter region. **(I)** Methylation of IKKβ in the coding region. **(J)** Methylation of PDYN in the coding region. **(K)** Methylation of PDYN in the promoter region. **(L)** Methylation of DRD2 in the promoter region. **(A-F)** at the two-hour timepoint, while (G-L) are at the 1 week time point. **(M)** Global DNA methylation 2 h after stress. **(N)** Global DNA methylation 1 week after stress. ‘$’ indicates a significant main effect of stress, ‘#’ indicates a significant main effect of sex, and ‘x’ indicates a significant stress by sex interaction by two-way ANOVA (*p* < 0.05). *n* = 3–7 per group.

In the NAc at the two-hour time point, there were no group differences in methylation of the coding region of NEMO (IKKγ), as the main effects of stress [*F*_(1, 22)_ = 0.991, *p* = 0.332] and sex [*F*_(1, 22)_ = 0.357, *p* = 0.557] as well as the stress-by-sex interaction [*F*_(1, 22)_ = 0.496, *p* = 0.49] did not reach significance (Figure 4A). For the NEMO promoter, a significant effect of sex was observed in which females exhibited higher levels of methylation than males [*F*_(1, 22)_ = 20.96, *p* < 0.001; Figure 4B], but neither the main effect of stress [*F*_(1, 22)_ = 0.453, *p* = 0.509] nor the main effect of sex [*F*_(1, 22)_ = 0.391, *p* = 0.539] were statistically significant. A significant stress-by-sex interaction was observed for methylation in the coding region of IKKβ, [*F*_(1, 22)_ = 5.62, *p* = 0.028; Figure 4C]. Although stress did not significantly affect methylation in either sex, the insignificant ‘trends’ it induced were in opposite directions: increasing methylation in females (*p* = 0.062) and slightly reducing it in males (*p* = 0.197). There was no overall main effect of sex [*F*_(1, 22)_ = 0.281, *p* = 0.602] or stress [*F*_(1, 22)_ = 0.364, *p* = 0.553]. For the coding region of PDYN, there was a significant main effect of stress [*F*_(1, 22)_ = 6.28, *p* = 0.021; Figure 4D], in which 5DS reduced methylation. However, neither the effect of sex [*F*_(1, 22)_ = 0.239, *p* = 0.631] nor the stress-by-sex interaction [*F*_(1, 22)_ = 0.044, *p* = 0.836] were significant. There were no group differences in methylation of the PDYN promoter (Figure 4E), as the effects of stress [*F*_(1, 22)_ = 2.199, *p* = 0.155], sex [*F*_(1, 22)_ = 2.329, *p* = 0.143], and their interaction [F_(1, 22)_ = 2.727, *p* = 0.115] all failed to reach significance. Finally, there was a significant main effect of stress on methylation in the coding region of Dopamine Receptor D2 (DRD2) [*F*_(1, 22)_ = 15.53, *p* < 0.001; Figure 4F] in which 5DS significantly increased methylation at this site. However, the effect of sex [*F*_(1, 22)_ = 2.304, *p* = 0.145] and the stress-by-sex interaction [F_(1, 22)_ = 1.142, *p* = 0.299] were both insignificant.

At the one-week time point following stress in the NAc, no group differences were observed for the coding region of NEMO (Figure 4G), as the main effects of stress [*F*_(1,18)_ = 0.423, *p* = 0.525] and sex [*F*_(1,18)_ = 0.371, *p* = 0.552] were insignificant, as was the stress-by-sex interaction [*F*_(1,18)_ = 0.335, *p* = 0.571]. Similarly, the effects of stress [*F*_(1,18)_ = 0.188, *p* = 0.671], sex [*F*_(1,18)_ = 0.441, *p* = 0.517], and their interaction [*F*_(1,18)_ = 3.995, *p* = 0.064] were also not significant for IKKβ (Figure 4I). However, a significant main effect of sex was observed for NEMO promoter methylation [*F*_(1,18)_ = 23.5, *p* < 0.001; Figure 4H] in which females exhibited more methylation than males. The main effects of stress [*F*_(1,18)_ = 0.027, *p* = 0.872] and the stress-by-sex interaction [*F*_(1,18)_ = 0.019, *p* = 0.892] were not significant for NEMO promoter methylation. A significant main effect of stress [*F*_(1,18)_ = 4.94, *p* = 0.042; Figure 4J] and stress-by-sex interaction [*F*_(1,18)_ = 5.37, *p* = 0.035; Figure 4J] were observed for the coding region of the prodynorphin gene. For this interaction, PDYN methylation was increased by stress in female mice (*p* = 0.01) but not in males (*p* = 0.941). There was no significant main effect of sex [*F*_(1,18)_ = 1.206, *p* = 0.289] for PDYN methylation, however. Regarding the promoter region of PDYN in the NAc, stress increased PDYN promoter methylation [*F*_(1,18)_ = 4.738, *p* = 0.046], but the effect of sex [F_(1,18)_ = 1.323, *p* = 0.268] and the stress-by-sex interaction [*F*_(1,18)_ = 0.012, *p* = 0.915] did not reach statistical significance (Figure 4K). No significant effects of stress [*F*_(1,18)_ = 0.647, *p* = 0.434], sex [*F*_(1,18)_ = 2.088, *p* = 0.169], or their interaction [*F*_(1,18)_ = 1.593, *p* = 0.226] were observed for DRD2 methylation in the NAc (Figure 4L).

In the NAc, global methylation analysis revealed no significant effects of stress [*F*_(1, 22)_ = 0.025, *p* = 0.875] or sex [*F*_(1, 22)_ = 1.709, *p* = 0.207] on DNA methylation at the two-hour timepoint (Figure 4M). The stress-by-sex interaction was also not significant [*F*_(1, 22)_ = 0.607, *p* = 0.446]. However, at the one-week timepoint, a significant main effect of stress was observed in which the stress-exposed animals had less methylation overall compared to controls [*F*_(1, 18)_ = 13.748, *p* = 0.002; Figure 4N]. The stress-by-sex interaction was not significant, however, [*F*_(1, 18)_ = 2.141, *p* = 0.164], and there was no main effect of sex [*F*_(1, 18)_ = 0.126, *p* = 0.727].

**Figure 5:**
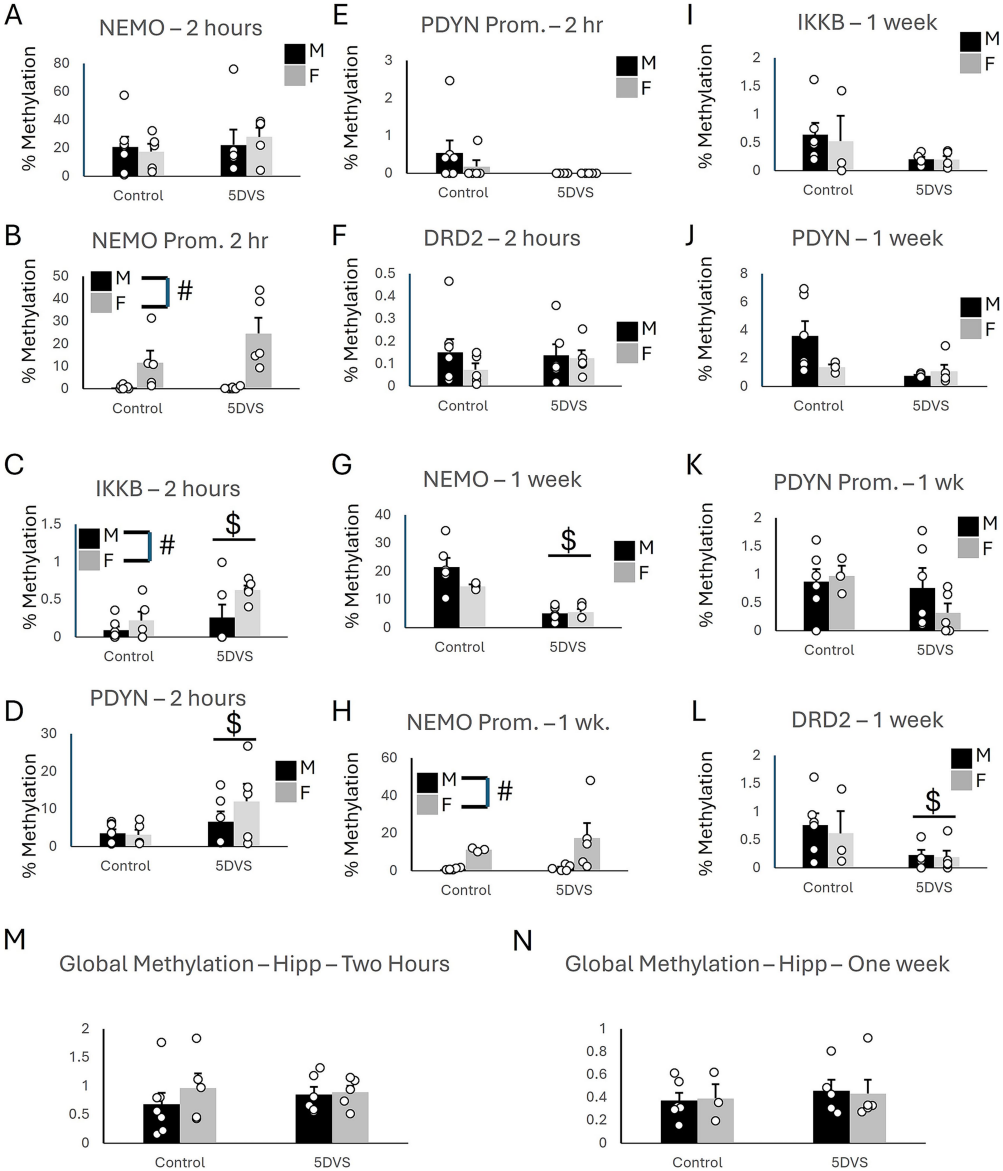
Epigenetic consequences of stress in the hippocampus. **(A)** Methylation of NEMO in the coding region. **(B)** Methylation of NEMO in the promoter region. **(C)** Methylation of IKKβ in the coding region. **(D)** Methylation of PDYN in the coding region. **(E)** Methylation of PDYN in the promoter region. **(F)** Methylation of DRD2 in the promoter region. **(G)** Methylation of NEMO in the coding region. **(H)** Methylation of NEMO in the promoter region. **(I)** Methylation of IKKβ in the coding region. **(J)** Methylation of PDYN in the coding region. **(K)** Methylation of PDYN in the promoter region. **(L)** Methylation of DRD2 in the promoter region. **(A-F)** at the two-hour timepoint, while (G-L) are at the 1 week time point. (M) Global DNA methylation 2 h after stress. (N) Global DNA methylation 1 week after stress. ‘$’ indicates a significant main effect of stress, and ‘#’ indicates a significant main effect of sex by two-way ANOVA (*p* < 0.05). *n* = 3–7 per group.

At the two-hour time point in the Hip, there were no significant group differences in methylation of the NEMO coding region (Figure 5A), as the effects of sex [*F*_(1, 22)_ = 0.018, *p* = 0.895], stress [*F*_(1, 22)_ = 0.509, *p* = 0.484], and the stress-by-sex interaction [*F*_(1, 22)_ = 0.301, *p* = 0.59] were all insignificant. However, a significant main effect of sex was observed on NEMO promoter methylation, in which females exhibited significantly more methylation than males [*F*_(1, 22)_ = 21.0, *p* < 0.001; Figure 5B]. No significant of stress was observed for NEMO promoter methylation [*F*_(1, 22)_ = 0.453, *p* = 0.509], and the stress-by-sex interaction was not significant either [*F*_(1, 22)_ = 0.391, *p* = 0.539]. Stress significantly increased methylation of both IKKβ [*F*_(1, 22)_ = 6.41, *p* = 0.020; Figure 5C] and PDYN [*F*_(1, 22)_ = 5.13, *p* = 0.035; Figure 5D] coding regions. A significant main effect of sex was also observed for IKKβ [*F*_(1, 22)_ = 4.76, *p* = 0.042; Figure 5C] in which females had more methylation than males. However, there was no significant stress-by-sex interaction for IKKβ [*F*_(1, 22)_ = 1.079, *p* = 0.312; Figure 5C]. For the PDYN coding region, the main effect of sex was not significant [*F*_(1, 22)_ = 0.872, *p* = 0.362], and neither was the stress-by-sex interaction [*F*_(1, 22)_ = 1.193, *p* = 0.288; Figure 5D]. There were no group differences in methylation of the PDYN promoter (Figure 5E), as the main effects of stress [*F*_(1, 22)_ = 2.609, *p* = 0.123] and sex [*F*_(1, 22)_ = 0.685, *p* = 0.418] and the stress-by-sex interaction [*F*_(1, 22)_ = 0.685, *p* = 0.418] were all not significant. The main effects of stress [*F*_(1, 22)_ = 0.143, *p* = 0.710] and sex [*F*_(1, 22)_ = 0.853, *p* = 0.367] on DRD2 coding region methylation were not significant, nor was the stress-by-sex interaction [*F*_(1, 22)_ = 0.438, *p* = 0.516; Figure 5F] in the Hip.

In the Hip at the one-week time point, a significant main effect of stress was observed for methylation in the coding region of NEMO [*F*_(1,18)_ = 29.27, *p* < 0.001; Figure 5G] in which 5DVS significantly reduced methylation. However, no significant main effect of stress [*F*_(1,18)_ = 1.898, *p* = 0.189] was observed, and the stress-by-sex interaction was not significant for NEMO methylation [*F*_(1,18)_ = 2.3, *p* = 0.15]. Females were observed to exhibit significantly more methylation of the NEMO promoter than males [*F*_(1,18)_ = 8.42, *p* = 0.011; Figure 5H], but no effects of stress were observed on the NEMO promoter [*F*_(1,18)_ = 0.563, *p* = 0.465], and no stress-by-sex interaction was observed [*F*_(1,18)_ = 0.385, *p* = 0.544]. Methylation of IKKβ did not differ significantly between the groups, as the main effects of stress [*F*_(1,18)_ = 3.572, *p* = 0.078] and sex [*F*_(1,18)_ = 0.094, *p* = 0.764] and their interaction [*F*_(1,18)_ = 0.075, *p* = 0.788] failed to reach statistical significance (Figure 5I). Similarly, the effects of stress [*F*_(1,18)_ = 4.308, *p* = 0.056], sex [*F*_(1,18)_ = 1.61, *p* = 0.224], and the stress-by-sex interaction [*F*_(1,18)_ = 2.915, *p* = 0.108] were also not significant for methylation of the PDYN coding region (Figure 5J). No group differences were observed for the PDYN promoter (Figure 5K), as the main effects of stress [*F*_(1,18)_ = 1.727, *p* = 0.209] and sex [*F*_(1,18)_ = 0.022, *p* = 0.885] were not significant, and neither was the stress-by-sex interaction [*F*_(1,18)_ = 0.042, *p* = 0.841]. Finally, a significant 5DS-induced reduction in methylation was observed for the coding region of DRD2 [*F*_(1,18)_ = 29.27, *p* < 0.001; Figure 5L], but the effect of sex [*F*_(1,18)_ = 0.204, *p* = 0.658] and the stress-by-sex interaction [*F*_(1,18)_ = 0.062, *p* = 0.807] were not significant.

In the Hip, global methylation analyses revealed no significant effects of stress [*F*_(1, 22)_ = 0.68, *p* = 0.797] or sex [*F*_(1, 22)_ = 0.703, *p* = 0.412] at the two-hour time point (Figure 5M). The stress-by-sex interaction was also not significant at the two-hour time point [*F*_(1, 22)_ = 0.425, *p* = 0.522]. Similarly, no significant main effects of stress [*F*_(1,18)_ = 0.387, *p* = 0.543] or sex [*F*_(1,18)_ = 0.001, *p* = 0.978] were observed at the one-week time point, and the stress-by-sex interaction also failed to reach significance [*F*_(1,18)_ = 0.043, *p* = 0.838; Figure 5N]. The methylation results are summarized in Table 2.

**Table 2:** Main effects of epigenetic consequences in the nucleus accumbens and the hippocampus.

Gene	NAc (2 h)	Hip (2 h)	NAc (1 week)	Hip (1 week)
NEMO	-	-	-	M. E. of Stress, Stress-Induced ↓
NEMO Promoter	M. E. of Sex, ↑ in F	M. E. of Sex, ↑ in F	M. E. of Sex, ↑ in F	M. E. of Sex, ↑ in F
IKKβ	Stress x Sex Interaction	M. E.s of Sex and Stress, ↑ in F, Stress-Induced ↑	-	-
PDYN	M. E. of Stress, Stress-Induced ↓	M. E. of Stress, Stress-Induced ↑	M. E. of Stress, Stress x Sex Interaction, ↑ in F only, not M	-
PDYN Promoter	-	-	-	-
DRD2	M. E. of Stress, Stress-Induced ↑	-	-	M. E. of Stress, Stress-Induced ↓
Global Methylation	-	-	M. E. of Stress, Stress-Induced ↓	-

## 4 Discussion

The results of the current study indicate that female c57BL/6 mice exhibit a slight increase in stress susceptibility compared to males. Females have previously been reported to exhibit greater vulnerability than males to a six-day SCVS paradigm (Hodes et al., 2015), so our results are generally consistent with prior work. However, several studies using SCVS have reported sex differences of greater magnitude than those reported here, as studies often find that males are almost entirely resistant to the behavioral effects of SCVS (Hodes et al., 2015; LaPlant et al., 2009; Williams et al., 2020). In contrast, both the current and previous 5DS models our lab has used exert largely similar behavioral effects in males and females (Baugher et al., 2022). Given that 5DS and 5DVS are 1 day shorter than SCVS, and two of the three stressors used are identical across all three paradigms, we had not expected these shorter paradigms to have a greater effect on male behavior than SCVS. Other than being 1 day shorter, the only procedural differences between these models lie in the third stressor used, which consists of foot shocks in SCVS, forced swimming in 5DS, and fox urine exposure in 5DVS. Exposure to predator odor has also previously been reported to exhibit sex-specific effects, as trimethyl thiazoline (TMT, a major component of fox feces) has been reported to inhibit cellular proliferation in the hippocampus in males, but not females (Falconer and Galea, 2003), suggesting that males may be more sensitive than females to at least some of the consequences of predator odors. Similarly, there is some evidence that males may be more susceptible than females to forced swimming. Prior work in rats has shown that male rats exhibit a greater increase in immobility time following repeated exposure to forced swimming compared to females (Colom-Lapetina et al., 2017; Dalla et al., 2005). In our prior 5DS study, we essentially replicated this effect, as forced swimming was used as both a stressor and a behavioral test (Baugher et al., 2022). It is likely that the apparent increase in susceptibility to 5DS and 5DVS compared to SCVS in males is due to their increased sensitivity to forced swimming and predator odors, but other differences in animal husbandry, facilities, and genetic drift between labs could also contribute.

The only test in which females exhibited greater sensitivity to stress in the current study was the LDE. Effects of 5DVS in the OFT and ERC task did not differ significantly between the sexes, and no significant effects of 5DVS were observed in the EPM or FST. In contrast, the highly similar 5DS paradigm exerted significant effects in both the EPM and the FST. These results could be interpreted to suggest that forced swimming is perceived as more stressful than fox urine exposure. However, at least for the FST, this interpretation would be confounded by the fact that 5DS-exposed animals had prior experience with swimming. Indeed, it is likely that swimming experience rather than stress in general was likely the primary driver of the 5DS-induced behavioral change in the FST, although this would not explain the increased sensitivity of the EPM to 5DS over 5DVS. Although there are now numerous reports (including this one) that females exhibit increased stress susceptibility compared to males, it is important to emphasize that this is only true in specific instances, not universally. Indeed, growing evidence suggests that sex differences in stress responding are critically dependent on the exact combination and duration of stressors used, the behavioral tests being measured, and the time points at which behavior is examined (Hodes et al., 2015; LaPlant et al., 2009; Baugher et al., 2022; Baugher and Sachs, 2022; Eck et al., 2020; Duque-Wilckens et al., 2022; Johnson et al., 2020). Stress is known to impact a wide range of behavioral and cognitive domains, and it will be important for future research to examine potential sex differences in susceptibility to stress-induced changes in sleep, memory, feeding, attention, and social behaviors, as it is likely that different sex differences will be observed depending on the types of tasks used.

One ongoing issue in preclinical stress susceptibility research involves inherent difficulties in understanding the relevance of commonly used rodent behavioral paradigms for human psychopathology (Stupart et al., 2023; Gyles et al., 2023). One prominent controversy in this area deals with the interpretation of FST data. For example, immobility in the FST has been reported to reflect despair-like behavior (Porsolt et al., 1977), psychomotor retardation (Unal and Canbeyli, 2019), reduced anxiety (Anyan and Amir, 2018; Lee et al., 2017), and a passive stress-coping strategy (Commons et al., 2017). While there is likely merit in each of these interpretations in particular contexts, it remains difficult, if not impossible, to know whether increased immobility reflects increased despair, decreased anxiety, neither, or both. Similarly, observing no significant effects of stress in the FST, as was the case here, could indicate that stress did not impact any of these behavioral domains. Alternatively, a null result could stem from stress increasing panic/anxiety, which would promote swimming, while simultaneously promoting despair or locomotor retardation, which would reduce swimming. Whether the current null findings reflect two (or more) opposing behavioral modifications or a more straightforward lack of effect remains unclear. In addition, while significant behavioral effects of stress were observed in some tests (i.e., the LDE) but not others (e.g., EPM and FST), it is not clear whether the time point or the behavioral test is the main driver of these differential effects. Indeed, it is possible that different behavioral testing schedules would yield different results, but future research would be required to evaluate this.

Regardless, the development and validation of new behavioral measures that improve the translational relevance of preclinical research and align more clearly with RDoC-defined behavioral domains than traditional rapid behavioral tests could significantly enhance the impact of preclinical research related to psychopathology (Stupart et al., 2023; Gyles et al., 2023). The ERC test used in the current work is a potential example of such a behavioral assay. This task was based on similar tests in which animals are given the option between a high value/high effort reward and a low value/low effort reward. Most prior studies of effort-related decision making have been conducted outside of the home cage in operant chambers (Ecevitoglu et al., 2025; Floresco et al., 2008; Marangoni et al., 2023; Shafiei et al., 2012) or in mazes with rewards of different values and/or effort requirements placed in different arms (Salamone et al., 1994; Correa et al., 2016; Dieterich et al., 2020; Carratala-Ros et al., 2019). To our knowledge, the exact home-cage ERC task employed here has not been reported previously, but it has several advantages, including the fact that data can be tracked continuously over long periods under conditions that are extremely familiar to the animals. We acknowledge that data interpretation in this test is not entirely unambiguous as reductions in wheel running could result from a failure to experience the rewarding effect of running, from a lack of energy, from psychomotor retardation, or from a combination of these effects. However, any of these possibilities could have important implications for psychiatric conditions like major depression, and further characterizations of the factors that govern behavior in this test will help evaluate its validity.

Using the ERC instead of traditional sucrose preference tests has several potential benefits. First, it provides a potential measure of anhedonia that involves at least some motivational component. Anhedonia in humans is typically measured using rating scales that focus on motivational, ‘wanting’ aspects of anhedonia, rather than ‘liking’ aspects of anhedonia (Markov, 2022). The sucrose preference test is a direct measure of ‘liking,’ as it is equally easy to choose between the sweet and standard solutions. Importantly, sweet preference generally remains intact even in anhedonic patients (Berlin et al., 1998; Dichter et al., 2010; Scinska et al., 2004), and thus sucrose preference tests are likely focusing on an aspect of anhedonia that is distinct from what is most commonly observed clinically. Second, providing animals with an additional choice may also improve translational relevance, as it is rare that humans have a single binary reward option. Third, the inclusion of a running wheel provides an important element of enrichment and allows for behavioral testing to occur in less impoverished conditions than the standard sucrose preference test. However, given that running wheel access is known to lead to antidepressant-like effects (Bjornebekk et al., 2005; Warner et al., 2024), including the ERC among a panel of behavioral tests could potentially impact behavior in other tests. Our published and current data demonstrate that the sub-chronic stress paradigms we use in the lab (5DS and 5DVS) are insufficient to induce reductions in sucrose preference in either the traditional sucrose preference test or in the context of the ERC test (Baugher et al., 2022). Thus, running disruptions in the ERC may be more sensitive than traditional sucrose preference tests for detecting the effects of sub-chronic stress, although future research using other stress paradigms would be required to determine whether this potential increase in sensitivity is generalizable across paradigms.

Regarding the epigenetic analyses, our results suggest that methylation patterns vary widely across the sites examined here following stress (see Table 2). Based on prior studies reporting increased expression of Dnmt3a in the NAc of females compared to males (Hodes et al., 2015), we had hypothesized that higher levels of methylation would be observed in females in that brain region. In addition, our published 5DS study reported that stress increased Dnmt3a expression in females, but not males (Baugher et al., 2022), so we further hypothesized that stress would increase methylation to a greater extent in females as well. However, we observed an equal number of statistically significant increases and decreases in methylation following stress exposure in our methylation-specific PCR analysis, and our global methylation studies revealed a stress-induced decrease in methylation 1 week following stress exposure. While these findings were not consistent with our hypothesis, they reflect the complexity of epigenetic alterations that occur following stress across brain regions and time points.

Having performed our behavioral and epigenetic analyses on separate cohorts of mice, it was not possible to correlate methylation patterns with behavioral outcomes. Collecting tissue at the two-hour time point made it impossible to assess subsequent behavioral changes in those animals, as their brains were collected prior to the time at which the first behavioral test would have been conducted. It would have been possible to conduct behavioral testing on the mice examined at the 1 week time point, which would have enabled us to evaluate correlations between behavioral and molecular outcomes, but having animals engage in behavioral tasks prior to tissue collection at the 1 week time point (but not the 2 h time point) would make it impossible to determine whether any differences observed between the 1 week and 2 h time points were due to time itself or the experience of behavioral testing. Future research would be required to provide a more comprehensive time course of methylation changes induced by stress and to determine whether particular methylation changes correlate closely with specific behavioral outcomes.

Candidate genes for the current experiments were chosen based on our prior work documenting sex-specific effects of 5DS on these genes (Baugher et al., 2022), which we hypothesized could be differentially expressed due to alterations in methylation. Prior work had suggested that NfκB signaling is a critical driver of the increased susceptibility of female mice to sub-chronic stressors, and NEMO and IKKβ were among the genes most prominently implicated as potential determinants of susceptibility vs. resilience (LaPlant et al., 2009). Our findings of sex differences in methylation of both NEMO and IKKβ at baseline and following stress further suggest that these genes have the potential to play a role in the sex differences in stress susceptibility observed here, but additional research would be required to evaluate this. The most consistent and greatest magnitude effects were observed for increased methylation of the NEMO promoter in both brain regions of females at both time points. In contrast, no significant effects of sex were observed in the coding region of NEMO, although stress did significantly reduce methylation of NEMO’s coding region in the Hip at the one-week timepoint. This overall sex difference at the NEMO promoter likely results from the fact that NEMO is located on the X-chromosome, and promoters of genes on the inactive X chromosome are often hypermethylated in females (Sharp et al., 2011; Cotton et al., 2011; Hellman and Chess, 2007).

The observed alterations in PDYN methylation could also be relevant for the observed stress-induced behavioral changes. Indeed, dynorphins are also known to mediate some of the behavioral effects of stress (Bruchas et al., 2010), at least in part through the activation of kappa opioid receptors (KORs) (McLaughlin et al., 2006; McLaughlin et al., 2006). Conversely, KOR blockade has been reported to induce antidepressant-like effects (Carr et al., 2010). We have previously reported that sub-chronic stress increases PDYN expression, but that this effect was greater in males than females (Baugher et al., 2022). Here, our results suggest that stress impacts PDYN methylation similarly in males and females 2 h after the final stress exposure, but at the one-week time point, stress increases PDYN methylation in females, but not males. Whether these sex differences in the stress-related regulation of PDYN are involved in the somewhat distinct behavioral outcomes following stress is not known. Similarly, whether these PDYN differences would impact therapeutic responses to KOR antagonism remains to be addressed, but preclinical work does suggest that males are more sensitive to at least some antidepressant-like effects of KOR antagonism than females (Laman-Maharg et al., 2018).

Finally, dopaminergic neurotransmission has been heavily implicated in stress susceptibility, particularly in the mesolimbic reward circuit (Cao et al., 2010; Ortiz et al., 1996; Baik, 2020). However, recent work also suggests that dopamine receptor-expressing neurons in the hippocampus are also engaged in and control anxiety-like behaviors (Godino et al., 2025). Our results show that stress induces significant epigenetic alterations in the DRD2 gene in both the Hip and the NAc, but these effects were largely similar in the two sexes and therefore unlikely to underlie the subtle differences in behavioral outcomes following stress observed here in males and females. Regardless, our findings provide additional data supporting the ability of stress to impact the brain’s dopamine system, which could have important implications for stress- and dopamine-related behaviors.

Two genes in the NAc (IKKβ at the two-hour time point and PDYN at the one-week time point) exhibited methylation patterns in the coding region that were governed by statistically significant sex-by-stress interactions. For both, stress increased methylation in females but not males. Several main effects of stress without interactions were also observed, including a stress-induced reduction in PDYN methylation in the gene body at the two-hour time point. In addition, at the one-week time point, stress increased methylation of DRD2 while reducing methylation of PDYN. Interestingly, DRD2 and PDYN are expressed in largely non-overlapping populations of medium spiny neurons (MSNs) in the NAc. However, whether the observed stress-induced changes in methylation are occurring in the cell types in which these genes are typically expressed or repressed is not known. Further, whether this reflects a general pattern toward stress reducing methylation of genes expressed in D1-MSNs while increasing methylation of genes expressed in D2-MSNs at the one-week time point would require additional experimentation. It is also worth noting that the effects of stress on PDYN methylation were heavily dependent on time, as methylation was reduced by stress at the two-hour time point, but increased by stress at the one-week time point.

Unlike the NAc, no significant sex-by-stress interactions were observed for DNA methylation in the Hip. However, both the NEMO promoter and the IKKβ gene body were shown to exhibit overall sex differences, with the NEMO promoter being hypermethylated in females while IKKβ was hypomethylated in females. In addition, stress significantly increased the methylation of IKKβ and PDYN at the two-hour time point while reducing methylation of NEMO and DRD2 at the one-week time point. Although no genes were shown to be significantly altered by stress at both time points, both IKKβ and PDYN were significantly upregulated by stress after 2 h but trended toward being reduced by stress at the one-week time point. These observations highlight the importance of time point when determining the epigenetic consequences of stress.

Taken together, males and females exhibited both similarities and differences in their behavioral and epigenetic responses to stress. Behaviorally, a significant sex difference in stress susceptibility was only observed in one test (the LDE), and only two sex-by-stress interactions were observed in our molecular analyses. In addition, only one overall sex difference in methylation was noted other than for the promoter of NEMO, an X-linked gene. Nonetheless, identifying sex differences in behavioral and molecular responses to stress allows for the possibility of testing the functional significance of these molecular alterations to gain insight into the biological basis of sex differences in stress susceptibility. Future research should continue to address this important area by determining whether stress-induced behavioral dysfunction can be treated more optimally by taking into account sex-specific molecular pathology.

## Data Availability

The raw data supporting the conclusions of this article will be made available by the authors, without undue reservation.
